# Larotrectinib in Mismatch-Repair-Deficient TRK Fusion-Positive Metastatic Colon Cancer After Progression on Immunotherapy

**DOI:** 10.7759/cureus.26648

**Published:** 2022-07-07

**Authors:** Pashtoon M Kasi, Maaz Khan Afghan, Andrew M Bellizzi, Carlos HF Chan

**Affiliations:** 1 Oncology, Weill Cornell Medicine, New York, USA; 2 Oncology, Bolan Medical College Complex Hospital, Quetta, PAK; 3 Pathology, Holden Comprehensive Cancer Center, University of Iowa, Iowa City, USA; 4 Surgery, Holden Comprehensive Cancer Center, University of Iowa, Iowa City, USA

**Keywords:** ntrk fusion, pembrolizumab, mismatch repair deficiency, microsatellite instability, liquid biopsy, larotrectinib, ipilimumab, colorectal cancer, circulating tumor dna

## Abstract

A 43-year-old woman presented with recurrent metastatic colon cancer with metastases to the peritoneum after having initially been diagnosed with stage IIB colon cancer and deferring adjuvant chemotherapy. Circulating tumor DNA (ctDNA)-based liquid biopsy testing revealed microsatellite instability-high (MSI-H) status, which was also confirmed on tissue testing. This patient then underwent four cycles of pembrolizumab and two cycles of ipilimumab and nivolumab (CTLA-4 rescue) with, unfortunately, progression of the disease. The patient was subsequently treated with larotrectinib, given the findings of TRK fusion-positive cancer on next-generation sequencing (NGS), and she was able to undergo curative surgery two months later that showed complete pathologic response. She continues to have no evidence of disease years later as well as no detectable ctDNA on NGS as well as tumor-informed minimal residual disease platforms. This case represents a marked and durable response to larotrectinib in a patient with deficiency in mismatch repair/MSI-H metastatic colorectal cancer harboring an *NTRK *fusion, bringing to light the potential for use of larotrectinib in earlier treatment lines in patients, and/or choice of targeted therapy versus immunotherapy in this patient subset.

## Introduction

In 2021, cancers of the colon or rectum are anticipated to account for nearly 150,000 diagnoses and over 50,000 cancer-related deaths [1]. The presence of genomic alterations in colorectal cancer (CRC) is a well-documented phenomenon, and universal testing for mismatch repair (MMR) or microsatellite instability (MSI) status is recommended, as the results of this test are useful for predicting the efficacy of pembrolizumab and nivolumab treatment [2]. Further, in the setting of advanced/metastatic disease, assessing the genomic status of Kirsten rat sarcoma virus (RAS) oncogene (*KRAS*), neuroblastoma RAS (*NRAS*), v-raf murine sarcoma viral oncogene homolog B1 (*BRAF*), human epidermal growth factor receptor 2 (*HER2*), and neurotrophic tyrosine receptor kinase (*NTRK*) is also recommended for determining treatment choice and eligibility for clinical trials of new therapeutics [2].

The incidence of microsatellite instability-high (MSI-H) status ranges from about 22% in stage II CRC to around 5% in stage IV tumors [3-5]. In stage II disease, a deficiency in MMR (dMMR) or an MSI-H status is a prognostic indicator of a favorable outcome [6], and in advanced disease, dMMR/MSI-H status predicts the efficacy of immunotherapy [7-9].

Tumor-agnostic drugs, or therapies targeting a biomarker of disease regardless of etiology, have generated great recent interest with the US Food and Drug Administration (FDA) approval of pembrolizumab, an anti-PD-1 antibody in 2017 for patients with metastatic cancers harboring dMMR/MSI-H solid tumors agnostic of tumor origin, and the approvals of larotrectinib in 2018 and entrectinib in 2019 for patients with advanced solid tumors harboring an *NTRK *fusion [10-12]. On June 16, 2020, pembrolizumab was also approved for tumor mutation burden high disease (TMB-high; ≥10 mutations/megabase (mut/Mb) on an FDA-approved companion diagnostic test) [11]. Of note, metastatic CRC (mCRC) patients harboring gene fusions have a more aggressive natural history compared to those patients without gene fusions [13]. The fusions of note tend to be enriched in dMMR/MSI-H mCRC.

*NTRK *fusions tend to be common events in rare tumor types and rare events in common tumor types [14]. The incidence of *NTRK *fusions in CRC is a rare phenomenon, with rates of 0.20% to 1%, but increases in MSI-H tumors to around 5% [15-18]. Interestingly, as noted, the incidence of dMMR/MSI-H status in kinase fusions is enriched, around 57%, with rates specifically in CRC patients harboring *NTRK *fusions from 62% to 88% [13,17,19], eliciting the support for screening dMMR/MSI-H patients for their *NTRK*-fusion status. 

## Case presentation

A 43-year-old female patient presented to an outside hospital in April 2018 with right lower quadrant (RLQ) pain, and computed tomography (CT) imaging showed abnormal thickening of the cecum and an adjacent abscess, compatible with perforated appendicitis. She underwent abscess drainage in April and an interval appendectomy that was converted to her right hemicolectomy with segmental small-bowel resection in June 2018. Pathology revealed an invasive adenocarcinoma of the cecum, and the patient was diagnosed with stage IIB (pT4aN0) disease, with intact MMR. She was offered adjuvant chemotherapy, which she declined.

**Figure 1:**
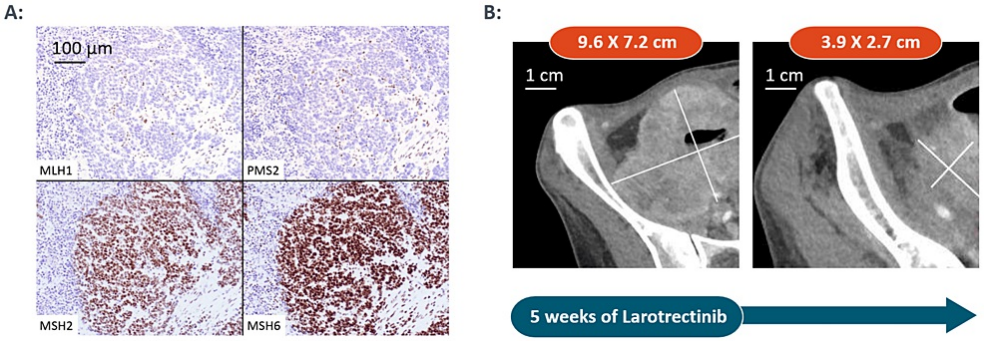
A. Immunohistochemical stains showing mismatch repair deficiency in our patient with colon (cecum) cancer; B. Imaging with computed tomography showing remarkable response to therapy with the tyrosine receptor kinase inhibitor

The patient received no further follow-up until June 2019, when she presented to our clinic with worsening abdominal pain. CT imaging showed recurrence of the RLQ mass, carcinomatosis, and ascites consistent with stage IV recurrent/metastatic colorectal (CRC) cancer. Circulating tumor DNA (ctDNA) testing of note revealed MSI-H. The patient’s MMR status was also re-evaluated from archived tissue and confirmed via immunohistochemistry (IHC) to have abnormal MMR proteins. Antibody testing for components of the MMR system showed no expression of mutL homolog 1 (MLH1) and postmeiotic segregation increased homolog 2 (PMS2) protein system in tumor nuclei, while normal adjacent tissue showed intact staining. IHC for mutS homologs 2 and 6 (MSH2/6) showed normal expression. Taken together, these results suggest a deficiency in the MMR system. IHC test was conducted by the Immunopathology Laboratory at the University of Iowa Hospitals and Clinics, on formalin-fixed tumor section using the following monoclonal antibodies: MLH1 clone ES05, PMS2 clone EP51, MSH2 clone FE11, and MSH6 clone EP49. All antibodies were used together with a polymer-based detection system. These results were discrepant with the interpretation rendered based on initial diagnostic material from the outside center (Figure 1A).

Given the incidence of *NTRK *fusions in MSI-H CRC, pathology also ordered pan-TRK for this patient, which is an IHC screening test for the overexpression of TRK, a family of proteins encoded by *NTRK* genes. Subsequent next-generation sequencing (NGS) confirmed that this patient did have a *TPR-NTRK1* gene fusion, and the tumor mutational burden (TMB) was also noted to be higher than 10 mutations/Mb (39 mutations/Mb) [11]. The tumor was also *RAS/RAF*-wild-type.

She was started on pembrolizumab in June 2019 (four cycles between June and August) but progressed clinically. Upon CT imaging in August 2019, it was identified that there was an interval increase in right pelvic mass, increased partial small bowel obstruction and invasion into the right iliopsoas muscle and encasement of the right external iliac vessels. At this point, she was placed on the combination immunotherapy regimen of ipilimumab and nivolumab therapy (CTLA-4 rescue; two cycles in September 2019). Clinically as well as on her CT scan in October 2019 there was a progression, with a mild interval increase in the size of the right pelvic locally recurrent tumor mass with stable metastatic RLQ mesenteric lymphadenopathy. At this point, after exhausting possible treatment with immunotherapy agents (pembrolizumab, and a combination of ipilimumab and nivolumab), she was placed on larotrectinib in November 2019, which specifically targets cancers harboring *NTRK *fusions.

After five weeks of treatment with larotrectinib, CT chest-abdomen-pelvis (CAP) revealed a considerable decrease in the size of previously noted largest peritoneal metastasis in the RLQ, along with a slight interval decrease in the size of previous mesenteric lymphadenopathy and an interval decrease in the degree of small bowel distention (Figure 1B). Of note, she clinically improved within one week of starting larotrectinib, with decreased pain and weight gain, which was remarkable.

**Figure 2:**
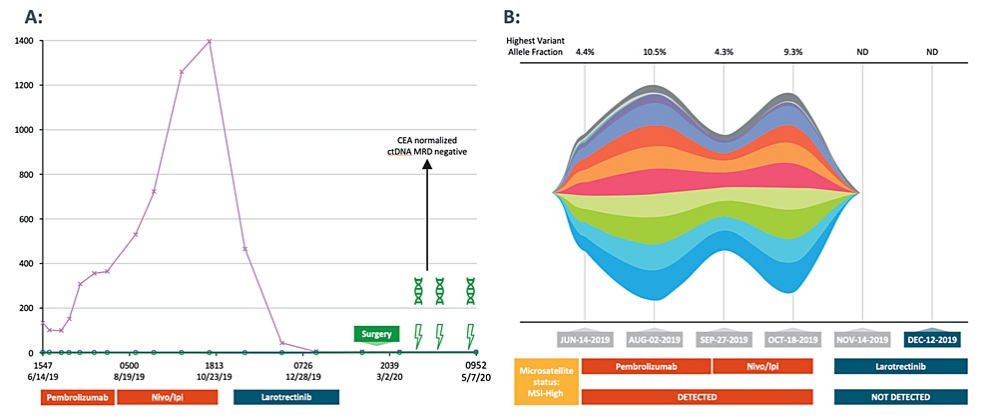
A. Serial CEA and tumor-informed ctDNA MRD testing in our patient with TRK fusion-positive colon cancer; B. Serial NGS-based ctDNA and plasma-MSI testing in our patient with TRK fusion-positive colon cancer CEA, carcinoembryonic antigen; ctDNA, circulating tumor DNA; Ipi, ipilimumab; MRD, minimal residual disease; MSI, microsatellite instability; ND, not detected; NGS, next-generation sequencing; Nivo, nivolumab; TRK, tyrosine receptor kinase.

By February 2020, the patient was still on larotrectinib, and given the excellent clinical, biochemical (carcinoembryonic antigen decline; ctDNA not detectable), and radiographic improvement, the patient underwent curative-intent surgical resection of the tumor mass, which included cytoreductive surgery, bilateral salpingo-oophorectomy, small bowel resection, omentectomy, tumor ablation of the small bowel, resection of peritoneal nodules, and resection of pelvic nodules. Her peritoneal carcinomatosis index was 8. Complete cytoreduction was achieved and hyperthermic intraperitoneal chemotherapy was deferred. All specimens were negative for carcinoma (complete pathologic response!). ‘Adjuvant’ TRK inhibitor to complete a total of six months of peri-operative therapy was discussed, and with markers including tumor-informed minimal residual disease (MRD) being negative, the patient elected for observation alone. To date ~26 months later, the patient continues to have no evidence of disease recurrence. Both NGS-based ctDNA testing including MSI-plasma as well as tumor-informed MRD ctDNA tests have been negative to date (Figure 2).

After surgery, the patient has been fully functional and back to work with excellent performance status and quality of life.

## Discussion

There are very limited, suboptimal treatment options for dMMR/MSI-H stage IV mCRC in patients after progression on immunotherapy. Compared to those with mismatch repair proficient/microsatellite stable (pMMR/MSS) disease, patients with dMMR/MSI-H mCRC show poorer outcomes on conventional chemotherapy [4,5]. Interestingly, recent data reveal a much greater likelihood of finding an NTRK fusion in CRC when testing the dMMR/MSI-H population [13,17,19,20]. The presented case supports clinical data from three pooled phase 1/2 trials, including the CRC tumor subgroup, that larotrectinib demonstrates a meaningful as well as a durable response, with a time to response of five weeks for this patient, in line with the 1.8 months as the median time to response reported in the pooled analysis [20,21]. This patient experienced rapid tumor progression on both first-line single-agent and dual-agent immunotherapy options.

Because molecular profiling had been performed upfront, upon arriving at our clinic in June 2019, we were able to identify the overexpression of TRK proteins, via Pan-Trk, an IHC screening assay, which can detect but not differentiate between *NTRK* wild-type and NTRK-fusions proteins. The presence of TPR-*NTRK1* -fusion transcript, an *NTRK *fusion, was confirmed following subsequent NGS testing. Our patient began treatment on larotrectinib after her CT scan showed progression on initially single and later combination immunotherapy.

There is a disparity in testing practices in the community setting, with just 40% of patients diagnosed with mCRC receiving guideline-aligned testing, and only 51% being tested for MSI/dMMR [22]. This case underlines the importance of adhering to guideline-recommended molecular testing practices and repeating IHC or MSI testing where there is high clinical suspicion as in our case (young female patient with large poorly differentiated right-sided colon cancer with lack of nodal metastasis). This patient’s *NTRK*-fusion status provided her with another option after she progressed on two immunotherapy options, which ultimately resulted in beneficial treatment response. Markers to date for dMMR/MSI-H mCRC refractory to immunotherapy are not known. In the landmark KEYNOTE-177 clinical trial leading to approval of pembrolizumab in first-line dMMR/MSI-H colon cancer, a third of the patients did not respond [20]. The unique nature of this patient’s phenotype brings into question the likelihood of successful treatment with immunotherapy in this patient population, especially given the risks for immune-related adverse events [23], supporting the further exploration of larotrectinib in the first-line setting in TRK fusion-positive dMMR/MSI-H mCRC.

## Conclusions

This case illustrates a patient with a unique phenotype who did not respond to both single-agent and combination immunotherapy until treating her *NTRK *fusion despite a high TMB. More importantly, the response to larotrectinib here was brisk, deep, and durable, with the patient having no evidence of disease ~26 months after surgery. This case illustrates the value of serial ctDNA (NGS and tumor-informed)-based testing with ctDNA kinetics and MRD testing as predictive markers of response to systemic therapy.
